# Hippocampal viscoelasticity and episodic memory performance in healthy older adults examined with magnetic resonance elastography

**DOI:** 10.1007/s11682-018-9988-8

**Published:** 2018-10-31

**Authors:** Lucy V. Hiscox, Curtis L. Johnson, Matthew D. J. McGarry, Hillary Schwarb, Edwin J. R. van Beek, Neil Roberts, John M. Starr

**Affiliations:** 1 Alzheimer Scotland Dementia Research Centre, University of Edinburgh, Edinburgh, UK; 2 Edinburgh Imaging Facility, Queens Medical Research Institute, University of Edinburgh, Edinburgh, UK; 3 Present Address: Department of Biomedical Engineering, University of Delaware, Newark, DE USA; 4 Thayer School of Engineering, Dartmouth College, Hanover, NH USA; 5 Beckman Institute for Advanced Science and Technology, University of Illinois at Urbana-Champaign, Champaign, IL USA; 6 Centre for Cognitive Ageing and Cognitive Epidemiology, Department of Psychology, University of Edinburgh, Edinburgh, UK

**Keywords:** Magnetic resonance elastography, Brain, Hippocampus, Cognition, Episodic memory, Viscoelasticity, Elastography

## Abstract

Episodic memory is particularly sensitive to normative aging; however, studies investigating the structure-function relationships that support episodic memory have primarily been limited to gross volumetric measures of brain tissue health. Magnetic resonance elastography (MRE) is an emerging non-invasive, high-resolution imaging technique that uniquely quantifies brain viscoelasticity, and as such, provides a more specific measure of neural microstructural integrity. Recently, a significant double dissociation between orbitofrontal cortex-fluid intelligence and hippocampal-relational memory structure-function relationships was observed in young adults, highlighting the potential of sensitive MRE measures for studying brain health and its relation to cognitive function. However, the structure-function relationship observed by MRE has not yet been explored in healthy older adults. In this study, we examined the relationship between hippocampal (HC) viscoelasticity and episodic memory in cognitively healthy adults aged 66–73 years (*N* = 11), as measured with the verbal-paired associates (VPA) subtest from the Wechsler Memory Scale (WMS-R). Given the particular dependence of verbal memory tasks on the left HC, unilateral HC MRE measurements were considered for the first time. A significant negative correlation was found between left HC damping ratio, ξ and VPA recall score (*r*_*s*_ = −0.77, *p* = 0.009), which is consistent with previous findings of a relationship between HC ξ and memory performance in young adults. Conversely, correlations between right HC ξ with VPA recall score were not significant. These results highlight the utility of MRE to study cognitive decline and brain aging and suggest its possible use as a sensitive imaging biomarker for memory-related impairments.

## Introduction

Age-related cognitive decline affects an estimated 40% of an otherwise healthy population over the age of 60 and reduces both quality of life and independent living (Small 2002). Episodic memory, which refers to the conscious recollection of a personal experience, is particularly sensitive to cerebral aging (Kinugawa et al. 2013), is more severely affected by age than other forms of memory (Levine et al. 2002), and is often the first and most prominent neuropsychological domain altered by Alzheimer’s disease (AD) (Albert et al. 2001; Bondi et al. 1999).

Episodic memory has long been recognised as being dependent on the function of an intact hippocampus (HC), a medial temporal lobe structure essential for encoding and consolidating new memories (Aggleton and Brown 1999; Eichenbaum et al. 2007); Vargha-Khadem et al. 1997). To study this relationship, many researchers typically rely on structural magnetic resonance imaging (MRI) to investigate the association between hippocampal volume (i.e. macroscopic size) and neuropsychological assessments of memory performance (Ferguson et al. 2010). The implicit link is that volume loss impairs function; however, measures of volume alone are not specific to the microstructural tissue alterations expected to impact memory function. As such, the conclusion from a large meta-analysis that the relationship between hippocampal size and episodic memory in normal aging is weak is perhaps not surprising (Van-Petten 2004). Instead, imaging techniques sensitive to the microscale characteristics of neural tissue are critically needed to better understand the origins of cognitive decline. Understanding how changes in hippocampal microstructure impact cognition in the context of aging may prove important for identifying critical neural underpinnings of the cognitive aging process and intervention targets for combating cognitive decline.

Over the last several years, magnetic resonance elastography (MRE) (Muthupillai et al. 1995) has emerged as a potentially useful clinical neuroimaging technique within the disciplines of radiology, neurology, and neurosurgery (Hiscox et al. 2016) due to its uniquely sensitive contrast mechanism (Mariappan et al. 2010). Unlike traditional magnetic resonance imaging (MRI), MRE provides a quantitative measurement of the mechanical properties (e.g. stiffness or viscosity) of the brain. Through recent advances in MRE technology, highly repeatable in vivo measurements of viscoelasticity have been reported for individual brain structures and regions (Johnson et al. 2016; Murphy et al. 2013). Previous MRE investigations have found that brain viscoelasticity is affected by neurodegeneration (Murphy et al. 2017) and intracranial tumours including cerebral malignancies (Hughes et al. 2015; Pepin et al. 2015). Adolescent children (McIlvain et al. 2018) and older adults (Hiscox et al. 2018) also show mechanical property regional variation compared to young adults, suggesting possible developmental trajectories for viscoelastic properties. Emerging evidence suggesting that mechanical signals operate in tandem with biochemical cues to determine tissue characteristics (Chanet and Martin 2014) indicates that brain viscoelasticity may provide novel information related to the underlying integrity of neural tissue microstructure (Sack et al. 2013). Through animal models of disease, the mechanical properties from MRE have been directly linked to demyelination (Schregel et al. 2012) and inflammation processes (Riek et al. 2012), as well as alterations in neuronal density (Freimann et al. 2013; Klein et al. 2014).

The relationship of brain viscoelasticity to the underlying neural microstructure (Sack et al. 2013) has also motivated the use of MRE for exploring brain-behavior (i.e. structure-function) relationships within cognitive neuroscience. In particular, several studies have investigated MRE-derived hippocampal viscoelasticity and its relation to cognitive function (Johnson et al. 2018; Sandroff et al. 2017; Schwarb et al. 2016, 2017). In 20 young healthy adults (aged between 18 and 33 years), results revealed a strong correlation between the relative viscous-to-elastic behavior (i.e. damping ratio, ξ) of the HC and relational memory (Schwarb et al. 2016). Of interest, measures such as hippocampal volume and metrics from diffusion tensor imaging (DTI) were not associated with memory performance. This work was later replicated across a larger sample (*N* = 51, aged between 18 and 35 years), and further demonstrated that higher aerobic fitness was associated with hippocampal ξ, which is interpreted to have mediated the benefits of fitness on memory performance (Schwarb et al. 2017). Subsequently, using a subsample of this population, a significant double dissociation between the orbitofrontal cortex-fluid intelligence relationship and the hippocampal-relational memory relationship was observed (Johnson et al. 2018), thus highlighting the potential of using MRE to map brain mechanical properties with regard to specific cognitive functions. However, in these studies only healthy young adults were recruited; the impact of changes to hippocampal viscoelasticity in the context of age-related cognitive decline remains unexplored.

In the present study, we sought to examine the relationship between hippocampal viscoelasticity and performance on a verbal paired associates task (VPA) in cognitively healthy older men and women. The VPA task involves learning the association between two pieces of information, with test materials presented as either semantically related or unrelated word pairs. This binding of information is thought to rely heavily on the hippocampal formation (Eichenbaum and Cohen 2001), and thus verbal paired associate learning tasks have become emblematic of hippocampal function. As lesion deficit and functional MRI (fMRI) studies have provided evidence for a material-specific lateralization of function, with the dominant (usually the left) HC mediating verbal memory (Frisk and Milner 1990), and non-dominant (usually the right) HC mediating nonverbal or visual memory (Smith and Milner 1981), we explored the potentially unique contributions of left HC viscoelasticity to VPA recall score. Additionally, as simulation experiments have demonstrated how atrophy, and the concomitant increase in cerebrospinal fluid (CSF), can produce a systematic bias in MRE-based stiffness measurements (Murphy et al. 2013), we developed and applied a novel image analysis procedure to remove CSF voxels from the MRE measurements that is compatible with our current protocol. This issue is of critical importance in the study of aging, where, on average, older adults are expected to have smaller brain volumes and higher levels of CSF (Erber 2012). Consistent with previous MRE studies of relational memory in young adults, and the particular dependence of verbal memory tasks on the left HC, we hypothesised that left HC viscoelasticity would show a significant correlation with memory performance in right-handed older adults – in particular, we predicted that a relatively greater viscous-to-elastic hippocampus (higher damping ratio, ξ) will be associated with poorer performance on the VPA recall task.

## Methods and materials

### Participants

Twelve healthy older adult participants (aged 65–75 years) were recruited from the Join Dementia Research database; demographic information on this sample has been reported previously (Hiscox et al. 2018). All participants were English native speakers, were right-handed, and had no history of neurological or psychiatric episodes. No significant structural MRI abnormalities were reported by a consultant radiologist. The study met all criteria for approval from the National Health Service (NHS) Lothian Research Ethics Committee (15/SS/0219) and written informed consent was obtained from each participant prior to neuroimaging and neuropsychological assessment.

### Neuroimaging acquisition

MRI scanning was performed using a Siemens 3 T Verio whole-body MRI scanner with a 12- channel head receive coil (Siemens Medical Solutions; Erlangen, Germany). A high-resolution *T*_1_-weighted MPRAGE (Magnetization-Prepared Rapid Gradient Echo) sequence was obtained consisting of the following parameters: 1 mm isotropic voxels; TE = 2.97 ms; TR = 2400 ms; FOV = 240 × 240; acquired in a sagittal orientation. To elicit brain tissue displacements for MRE, a commercially available actuator (Resoundant; Rochester, MN, USA) was set to a single frequency of 50 Hz and a driving amplitude of 20%; the vibrations were generated by the active driver situated in the MRI equipment room and transferred through a pneumatic hose to a soft pad placed below the occipital portion of the head. This particular actuator design has been found to be acceptable to participants over a wide age range, as we have previously reported (Hiscox et al. 2018). The MRE acquisition employed a 3D multislab, multishot spiral sequence to capture high-resolution displacement data at an isotropic resolution of 1.6 mm (Johnson et al. 2014). Following iterative image reconstruction and data processing, complex, full vector displacement fields were generated for mechanical property estimation.

### MRE inversion

An octahedral shear strain-based SNR measure (OSS-SNR) was calculated to ensure sufficient data quality for stable inversion (McGarry et al. 2011). Nonlinear inversion (NLI) using a heterogenous viscoelastic finite element model (McGarry et al. 2012; Van Houten et al. 2001) was combined with soft prior regularization (SPR) of the HC to estimate the complex shear modulus, G* = G′ + iG′′, from the full vector MRE displacement data. The finite element property description was iteratively updated to match the model displacements to the measured displacement data. SPR uses readily available anatomical information to reduce variability in regional property estimates (McGarry et al. 2013) and has been shown to improve MRE measures in the hippocampus (Johnson et al. 2016). Region of interest (ROI) masks are defined and a regularization term penalises heterogeneity within the ROI, (see next section for HC ROI mask generation). An SPR weight of 10^−10^ was chosen to promote homogeneity (~15% variation across the regions) while allowing for reasonable convergence speed within 100 global NLI iterations. In regions outside of the SPR masks, the property estimates were smoothed using a 1.5 mm Gaussian kernel between global optimization iterations to maintain stability. The smoothing operation does not cross regional boundaries, which allows sharper definition of structure boundaries and reduces variability in measures potentially arising from partial volume effects or contamination from nearby regions of CSF. Next, maps of the complex shear modulus G* were reformulated in MATLAB to provide quantitative maps of damping ratio, ξ = G′′/2G′, and shear stiffness, μ = 2|G*|^2^/(|G*| + G′). Damping ratio, ξ, is a dimensionless quantity describing the relative attenuation level in the material. Higher ξ values indicate a more viscous fluid, while low ξ values indicate a more elastic solid. More viscous and frictional losses (i.e. higher ξ) suggest a less densely connected solid phase which may be indicative of a reduction in tissue integrity (Munder et al. 2017; Schwarb et al. 2017). Shear stiffness is directly related to wavelength in a viscoelastic solid (Manduca et al. 2001), where shear waves will propagate more quickly through a stiff material (corresponding to a longer wavelength) than through a softer material.

### Hippocampal mask generation

Left, right, and bilateral hippocampal (HC) masks were obtained via automatic segmentation of the *T*_1_-weighted images using the FreeSurfer image analysis suite (v.5.3) through the recon-all pipeline (Fischl 2012). Segmentation quality was visually assessed and manual adjustments were made when necessary. The MRE *T*_2_-weighted magnitude images were then co-registered to the structural *T*_1_-weighted MPRAGE through an affine transformation (12-degrees of freedom, tri-linear interpolation and a correlation ratio cost function) using the FLIRT tool within FSL (FMRIB Software Library v.6.0) (Jenkinson et al. 2012). The registration was optimised by using weighting volumes of the ventricles. The inverse of this transform was then calculated to register the HC mask from the anatomical *T*_1_ image into MRE space; a threshold of 95% was applied to the masks to reduce partial volume effects. FAST (FMRIB’s Automated Segmentation Tool), was used to extract CSF maps from the *T*_1_-weighted image (Zhang et al. 2001). The output CSF map is a partial volume map containing intensity values representing the proportion of CSF within each voxel [from 0 to 100%]. The resulting CSF map from participants was co-registered to their MRE data using the same inverse transform as previously described. The CSF maps were then binarized and multiplied by the original HC mask to generate new smaller masks without CSF. In other words, only voxels that contained 0% CSF were included in the final HC masks. These masks were then input into SPR with the same weighting parameter *α* = 10^−10^ within the NLI algorithm, as mentioned previously, and voxels with CSF within the masks were left as distributed properties with no SPR to allow voxel properties to update freely during the iterative reconstruction. Automated labelling based on a spatial probabilistic atlas was performed to obtain bilateral HC volumes; Estimated Total Intracranial Volume (eTIV) was used to normalize HC volume for participant head size (Buckner et al. 2004).

### Neuropsychological assessments

The Montreal Cognitive Assessment (MoCA) is a widely used 30-point assessment administered in approximately 10 min to screen for global cognitive impairment (Nasreddine et al. 2005). All participants were required to score > 26/30 on the MoCA to ensure normal cognitive function. The National Adult Reading Test (NART) was administered to measure full scale intelligence (Nelson and Willison 1991). The NART can be used as a proxy for premorbid intelligence since it has been shown to remain impervious to mild-to-moderate memory decline (McGurn et al. 2004). All participants also completed the Edinburgh Handedness Inventory (EHI) to measure handedness. Episodic memory was assessed by using the Verbal Paired Associates subtest (VPA) from the Wechsler Memory Scale-Revised (WMS-R) (Wechsler 1987). The VPA is one of the most widely used instruments for measuring explicit episodic memory (Uttl et al. 2002). In this study, only the immediate recall (VPA-IM) test scores are reported. Delayed recall data were collected, however, inter-subject variability was restricted due to the limited maximum score being 8/8. This VPA-IM test involves the examiner reading eight word pairs to the participant across three study test trials. The VPA pairs can be divided into four “easy” pairs (semantically related) and four “hard” pairs (semantically unrelated). After each presentation of the list of eight pairs, the first word is given by the examiner and the participant is required to provide its associate. The maximum score from the three trials was 24.

### Statistical analyses

All statistical analyses were performed with IBM SPSS Statistics for Mac, version 25.0.0 (IBM Corp., Armonk, N.Y., USA).

#### Identification of outliers

Due to significant kurtosis (|K| > 1.96, see next section) in the test of memory performance (i.e. the VPA), median absolute deviation (MAD) methods were used to detect statistical outliers (Hampel 1974; Leys et al. 2013). A conservative criterion of 3 times the MAD was used for outlier detection (Miller 1991). One participant was excluded at this stage due to scoring below the MAD, suggesting a lack of engagement in the task, or an undiagnosed memory disorder with the score being within the range of that expected for a patient diagnosed with Alzheimer’s disease (Wechsler 1987). However, MAD did not identify any significant outliers due to hippocampal viscoelastic measures. The analytic sample therefore included 11 older adults (mean age = 69.1 + 2.3 years, 6 female, 5 male).

#### Test of normality

The normal distribution of each variable was investigated by calculating both skewness and kurtosis for the purpose of calculating whether parametric or non-parametric correlations were warranted. In particular, we calculated both |S| (skewness/standard error), and |K| (kurtosis/standard error); under the null hypothesis of normality both are roughly normally distributed. Thus when |S| or |K| > 1.96, the skewness is significantly different from zero (*p* < 0.05) and non-parametric correlations are required.

#### Inclusion of covariables

Univariate correlation analyses were performed to identify whether any covariates of interest (including age (years), sex, NART full-scale IQ, and HC volume) were associated with any of the dependant variables. Due to the limited sample size, only covariables that reached significance were entered into the model in order to reduce the risk of model over-fitting (i.e. when a statistical model contains more parameters than can be justified by the data). Age and sex were of interest due to previous studies identifying a link between both variables and brain viscoelasticity (Arani et al. 2015; Sack et al. 2011; Hiscox et al. 2018), whereas the NART full-scale IQ was included due to previous reports of an association between brain structure and intelligence (Lange et al. 2010). HC volume was also measured, as is the procedure for diagnostic MRI volumetry, to investigate whether HC size was associated with VPA recall score. To maintain consistency with other studies, volumetric measurements were derived from FreeSurfer, as opposed to the size of the HC masks in MRE native space.

#### Correlations with memory performance

Spearman partial correlation coefficients*, r*_*s*_, were used to investigate how each HC measure – damping ratio, ξ, shear stiffness, μ and volume – correlated with VPA recall performance. Identical analyses were performed on the left, right and bilateral HC. Correlations were two-tailed with level of significance set at *p* < 0.05.

#### Comparing the significance of dependent correlations

Statistical differences between correlations were determined using Steiger’s *z-*test (Steiger 1980), which requires the computation of a *z*-score based on the sample size and the correlation coefficients to be compared (r_jk_ and r_jh_), along with the correlation of the unshared variable (r_kh_). Steiger’s method has been found to be superior to other tests comparing dependent correlation coefficients (e.g. Hotelling’s t-test or Williams’ modified t-test), particularly at small sample sizes [Meng et al. 1992]. By convention, *z* values greater than 1.96 are considered significant. Calculations were performed using a web utility provided by (Lee and Preacher 2013).Due to the sample size, we also obtained the confidence intervals (CI) for the correlation coefficients as computed by bootstrapping (1000 draws; 95% CI level; Bias corrected accelerated), which, if not overlapping with one-another, provides evidence supporting an effect at *p* < 0.05.

## Results

Descriptive statistics (mean, standard deviation, minimum/maximum values, population coefficient of variation [CV], |S|, and |K| for all study variables for the analytic sample of 11 subjects (6F/5M) are presented in Table 1. All MRE data reported refer to results obtained in MRE native space for each participant. The mean OSS-SNR score indicates high quality MRE displacement data, and the mean NART full-scale IQ indicates that the sample was intellectually high functioning. All participants were right-handed as determined by the EHI.

**Table 1:** Descriptive statistics for study variables

	Mean	SD	Min/Max	CV	|S|	|K|
Demographics						
Age (years)	69.1	2.3	66/72	3.3%	0.43	−0.66
NART-full scale IQ	123.4	4.25	115/128	3.4%	−1.16	−0.13
MoCA	28.3	1.73	26/30	6.1%	−0.56	−1.39
VPA immediate recall	20.7	1.95	17/24	9.4%	−0.34	0.23
MRE measures						
OSS-SNR*	5.79	1.54	4.19/8.37	26.5%	0.93	0.09
Damping ratio ξ						
Bilateral HC ξ	0.176	0.039	0.118/0.243	22.5%	1.15	−0.09
Left HC ξ	0.162	0.052	0.109/0.276	31.9%	2.01	1.08
Right HC ξ	0.186	0.038	0.126/0.249	20.3%	0.66	−0.11
Stiffness μ [kPa]						
Bilateral HC μ	2.86	0.35	2.17/3.38	12.2%	−0.98	0.05
Left HC μ	2.77	0.51	2.06/3.83	18.6%	0.91	0.27
Right HC μ	2.91	0.40	1.93/3.47	13.8%	−2.17	2.47
Volume [cm^3^]						
Bilateral HC Volume	8.20	1.17	6.94/10.46	14.3%	1.37	−0.09
Left HC Volume	4.02	0.66	3.17/5.16	16.5%	0.74	−0.73
Right HC Volume	4.17	0.53	3.61/5.29	12.7%	1.98	0.77

*OSS-SNR mean values were calculated over the entire brain mask

> 1.96 for |S| and |K| indicate a violation of the assumption of normality

*MoCA*, Montreal Cognitive Assessment; *NART*, National Adult Reading Test; *VPA*, verbal paired associates; *OSS-SNR*, octahedral shear strain signal-to-noise ratio; *HC*, hippocampus; *CV*, coefficient of variation

Due to a violation of the assumption of normality for three variables (left HC ξ, right HC μ, and right HC volume), non-parametric statistical tests were conducted in subsequent analyses. Univariate analyses on the covariables of interest and their relationship to the dependent variables are shown in Table 2**.** The relationship between age and VPA score was significant (*r*_*s*_ = −0.60, *p* = 0.05), resulting in age being the sole covariable included in further analyses.

**Table 2:** Relationships between statistical covariates and dependent variables

	Covariates			
	Age	Sex	NART IQ	HC Volume
Dependent variables				
VPA recall score	r_s_ = −0.60, *p* = 0.05*	*U* = −1.59, *p* = 0.11	*r*_s_ = 0.45, *p* = 0.16	*r*_s_ = −0.06, *p* = 0.87
Bilateral ξ	*r*_s_ = 0.38, *p* = 0.24	*U* = −0.46, *p* = 0.65	*r*_s_ = 0.01, *p* = 0.97	*r*_s_ = −0.17, *p* = 0.62
Left ξ	*r*_s_ = 0.09, *p* = 0.79	*U* = −0.18, *p* = 0.86	*r*_s_ = 0.12, *p* = 0.73	*r*_s_ = 0.24, *p* = 0.48
Right ξ	*r*_s_ = 0.48, *p* = 0.14	*U* = −1.10, *p* = 0.27	*r*_s_ = −0.01, *p* = 0.97	*r*_s_ = − 0.39, *p* = 0.24
Bilateral μ	*r*_s_ = −0.18, *p* = 0.59	*U* = 0.00, *p* = 1.00	*r*_s_ = −0.28, *p* = 0.40	*r*_s_ = 0.16, *p* = 0.65
Left μ	*r*_*s*_ = −0.44, *p* = 0.18	*U* = −0.18, *p* = 0.86	*r*_s_ = 0.01, *p* = 0.99	*r*_s_ = 0.34, *p* = 0.31
Right μ	*r*_s_ = 0.06, *p* = 0.86	*U* = −0.37, *p* = 0.72	*r*_s_ = −0.26, *p* = 0.45	*r*_s_ = 0.25, *p* = 0.47
Bilateral volume	*r*_s_ = 0.04, *p* = 0.91	*U* = 0.00, *p* = 1.00	*r*_s_ = 0.35, *p* = 0.29	N/A
Left volume	*r*_s_ = 0.07, *p* = 0.85	*U* = 0.00, *p* = 1.00	*r*_s_ = 0.28, *p* = 0.40	N/A
Right volume	*r*_s_ = −0.15, *p* = 0.66	*U* = −0.18, *p* = 0.86	*r*_s_ = 0.40, *p* = 0.22	N/A

*p < 0.05

*r*_s_, Spearman correlation coefficient; *U*, Mann-Whitney U test statistic

### Correlations between MRE/MRI and memory performance

**Fig. 1:**
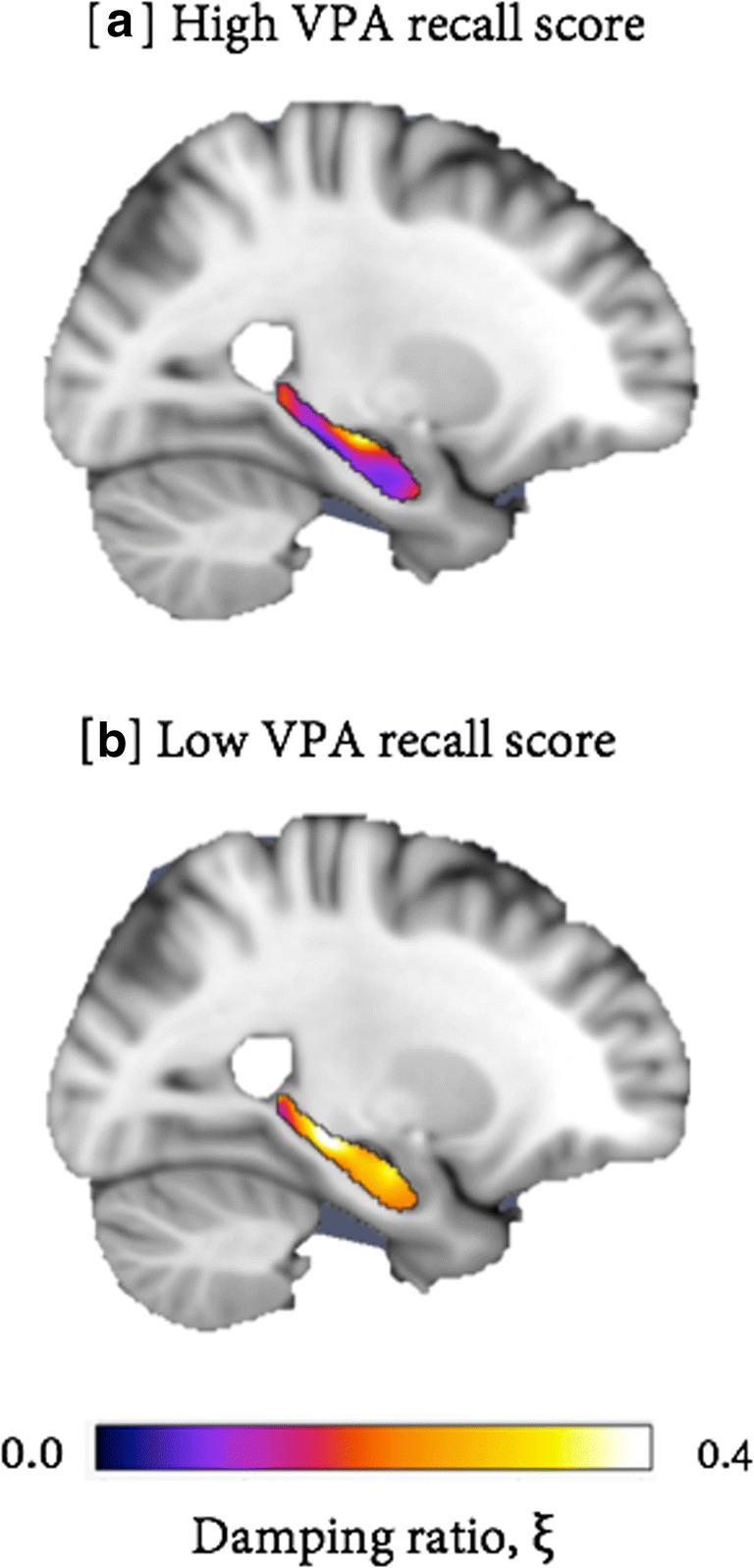
Example images of left HC damping ratio, ξ, for two participants. (**a**) Illustrates a participant who achieved a perfect score (24/24) on the VPA and possessed a more relatively elastic-to-viscous HC, whereas (**b**) shows a participant who scored 17/24 on the VPA and possessed a more relatively viscous HC. MRE information has been transformed to the standard *T*_1_ Montreal Neurological Institute (MNI152_1mm) template for illustration purposes

**Fig. 2:**
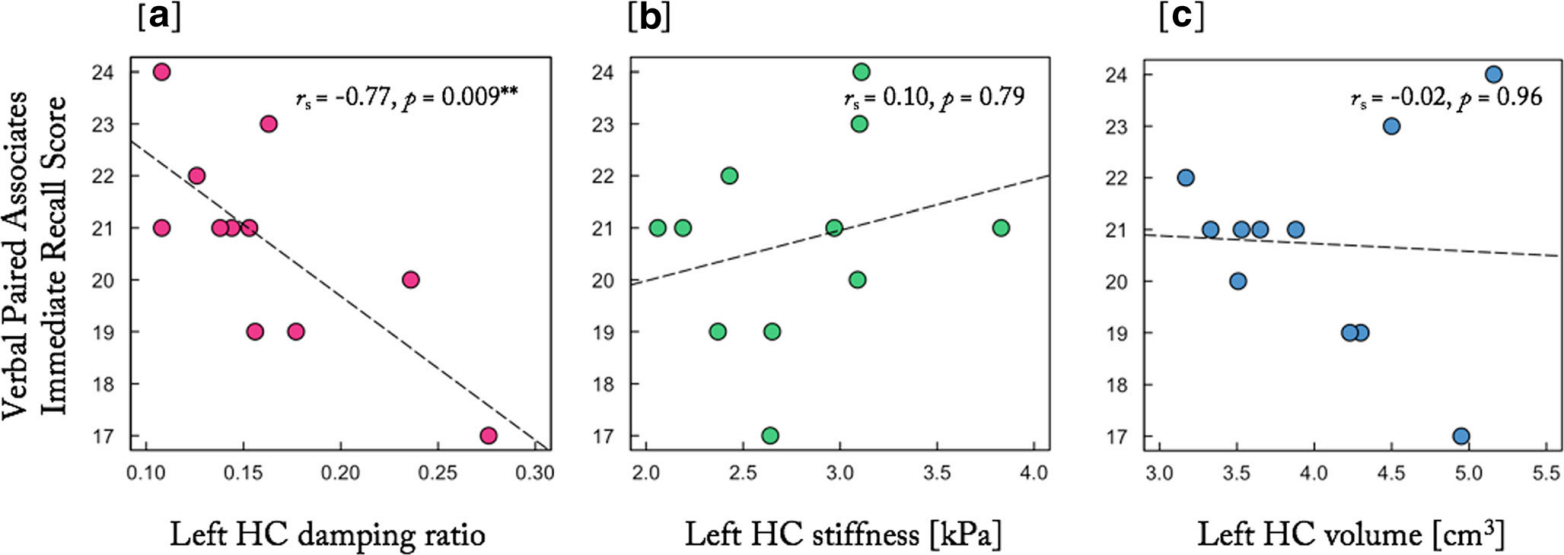
Left hippocampal (HC) structural metrics (**a**) damping ratio, ξ, (**b**) shear stiffness, μ, and (**c**) volume, plotted against episodic memory task performance; positive values indicate better task performance. Spearman non-parametric correlation, *r*_*s*_, demonstrates a significant negative correlation for HC ξ suggesting that greater viscous energy dissipation in the hippocampus indicated by high ξ is correlated with poorer performance in the individual’s episodic memory assessment. HC stiffness and volume plotted against VPA task performance, demonstrate no significant relationship with recall score. MRE data were collected at a 50 Hz vibration frequency

A significant negative correlation was found between left HC ξ and VPA recall score (*r*_*s*_ [8] = −0.77, *p* = 0.009, with 95% CI based on 1000 bootstraps of -0.93/-0.57). Figure 1 provides example MRE images of left HC ξ for both high and low VPA scores from two different participants. Analyses of left HC μ (*r*_*s*_ [8] = 0.10, *p* = 0.79) and left HC volume (*r*_*s*_ [8] = −0.02, *p* = 0.96) did not reveal any significant correlation with VPA score, as shown in Fig. 2.

Right HC ξ did not show any association with VPA score (*r*_*s*_ [8] = −0.51, *p* = 0.134 with 95% CI based on 1000 bootstraps of −0.91/+0.004), although this correlation was not statistically different from left HC ξ (*z* = 1.71, *p* = 0.09; r_jk_ = −.0.51, r_jh_ = −0.77, r_kh_ = 0.80). Also note that the 95% CIs for the correlation coefficients as computed by bootstrapping overlap for left and right HC ξ. Right HC μ and volume also showed no significant correlation with VPA score (ξ: *r*_*s*_ [8] = −0.51, *p* = 0.134; μ: *r*_*s*_ [8] = −0.36, *p* = 0.30; vol: *r*_*s*_ [8] = −0.01, *p* = 0.99). Nor was any significant relationship observed with bilateral HC measures (ξ: *r*_*s*_ [8] = −0.58, *p* = 0.077; μ: *r*_*s*_ [8] = −0.15, *p* = 0.68; vol: *r*_*s*_ [8] = −0.04, *p* = 0.91).

In addition, VPA score did not correlate with OSS-SNR measurements (*r* = 0.43, *p* = 0.17), and the OSS-SNR values did not correlate with any of the bilateral or unilateral MRE/MRI HC measurements (*p* > 0.05). Wilcoxon signed-rank tests revealed no significant differences between hemispheres for μ (z = −0.98, *p* = 0.33), whereas hemispheric differences were approaching significance for both ξ (z = −1.87, *p* = 0.06) and volume (z = −1.69, *p* = 0.09).

To investigate whether our regional specific analyses were warranted we also performed the same analysis with the global cerebrum as a reference region. Cerebral ξ was not significantly correlated with VPA recall score  (*r*_s_ = −0.04, *p* = 0.91 with 95% CI based on 1000 bootstraps of −0.49/+0.52); Steiger’s *z* test revealed this correlation was significantly smaller than the correlation observed between left HC ξ and episodic memory (*z* = 1.98, *p* = 0.048; r_jk_ = −0.04, r_jh_ = −0.77, r_kh_ = 0.03). Further, the 95% CIs for the correlation coefficients for left HC ξ and cerebral ξ do not overlap.

## Discussion

This preliminary investigation is the first to determine whether hippocampal (HC) viscoelasticity, measured *in vivo* using MRE, is associated with episodic memory performance in a group of healthy older adult participants. By adopting a processing pipeline that examined unilateral HC MRE measures and excluded voxels containing CSF, we have demonstrated that the relative viscous-to-elastic behavior (i.e. damping ratio, ξ) of the left HC is associated with explicit episodic memory, such that individuals with lower HC ξ performed better on the memory task. Right and bilateral HC ξ did not correlate with memory score, and neither did any of the HC volume or HC stiffness measurements.

Our results are consistent with previous work which has investigated the relationship between HC MRE and functional performance in healthy young adults (Johnson et al. 2018; Schwarb et al. 2016, 2017). The current study extends these findings by replicating this association in cognitively healthy older adults using a verbal-episodic memory task. However, it should be noted that in these previous studies, adjusted damping ratio, ξ’ = 1 – ξ, is reported, so that lower ξ’ would instead be indicative of a reduction in tissue integrity. These studies, like the present report, also did not find HC stiffness or HC volume to account for individual differences in memory. While smaller HC volumes, in general, tend to be associated with poorer memory performance, most studies that have investigated the relationship between size and memory has been in neurological patients, where smaller volumes are likely to be accompanied by other neuropathological features such as amyloid plaques and neurofibrillary tangles. In healthy aging, however, there appears to be little evidence for the “bigger is better” hypothesis (Van-Petten 2004); but see (Erickson et al. 2009, 2011), with there being substantial overlap between HC volume in healthy controls and patients with Alzheimer’s disease (Petersen 2004), and a large range of HC volumes in healthy adults (Barnes et al. 2004; Lupien et al. 2007). These results suggest that volume alone does not fully capture the extent of HC integrity, and smaller volumes may not necessarily signify deterioration. The large population variation found in this study for left HC ξ (32%), compared with left HC volume (17%), could suggest that MRE may be more sensitive for identifying the neural underpinnings of age-related cognitive decline within this older adult population.

All of the previous work highlighting the relationship between HC MRE and memory performance measured relational memory performance using a spatial reconstruction task. In the present study, participants were instead verbally presented with a list of word-pair items and after a very short delay were asked to provide the associate to a presented word. The current study took advantage of the VPA task from the WMS-R, a standard neuropsychological measure of episodic memory (Wechsler 1987). The VPA includes both an immediate and delayed test, though delayed recall measures were not considered in this report due to restricted inter-subject variability from limited test scores. While traditional views of HC function often emphasize that HC is necessary only after a delay e.g., (Baddeley and Warrington 1970; Smith and Milner 1981), the relational memory theory of HC function highlights the role of hippocampus in episodic binding of arbitrary relations across domains and delays (Cohen and Eichenbaum 1993; Eichenbaum and Cohen 2001; Monti et al. 2014). Therefore, the data presented in this work is consistent with findings demonstrating a relationship between HC structure and episodic memory with immediate recall only. Indeed, HC amnestic patients show impairment on relational memory tasks even at very short delays (Hannula et al. 2006; Horecka et al. 2018).

To our knowledge, unilateral HC MRE measurements have yet to be reported despite hemispheric asymmetries in the molecular and morphological characteristics of neuronal connections (Shipton et al. 2014); it has been suggested that unilateral specialisation may facilitate greater processing power by using the available neuronal circuitry more effectively. Our report of a significant correlation of ξ to VPA recall score the left HC only may be attributed to the role of the left HC in the storage of verbal material, as found in memory for immediate and delayed prose recall (Müller et al. 2005), free recall of word lists (Frisk and Milner 1990; Trenerry et al. 1993), and narratives (Milner 1971) as well as verbal memory, confrontation naming, and verbal conceptual ability (Seidenberg et al. 1998). In contrast, the right HC has been implicated in spatial and pictorial material, such as geometric faces and figures, not amenable to verbal processing (Gleissner et al. 1998; Leporé et al. 2009; Milner 1971), suggesting a functional hemispheric lateralisation of the right and left HC (Papanicolaou et al. 2002; Trenerry et al. 1993). In this study, however, we acknowledge that a single dissociation is not sufficient to demonstrate specificity for mapping cognitive function (Fama and Sullivan 2014), and future MRE studies may identify more precisely the cognitive functions supported by right HC ξ.

Previous work that has investigated the MRE-cognition relationship has been performed in young, healthy participants where soft-prior regularization (SPR) was deemed suitable to reduce partial volume effects and was shown to improve reliability and increase sensitivity of MRE measurements (Johnson et al. 2016). However, as normative aging studies generally reveal a decrease in overall brain volume, and a concomitant increase in CSF volume, we deemed that in this population a more conservative approach was required to minimize potential systemic biased caused by CSF. Acknowledging the bias caused by atrophy, (Murphy et al. 2013) created an MRE processing pipeline that utilized adaptive techniques to reduce edge artefacts due to the local homogeneity assumption required by direct inversion methods. This work demonstrated that the edge-related bias can be eliminated by eroding the ROI by 1 voxel from the brain’s surface. In the current study, we have instead used a heterogeneous inversion protocol and have proposed a processing pipeline that is specific for the removal of CSF present within the ROI itself. Removal of CSF from the HC masks prior to inversion is also likely to minimize the occurrence of quantitative errors due to data-model mismatch as a result of SPR enforcing CSF voxels to possess the same mechanical properties as solid tissue.

The damping ratio, ξ, dictates which component of the complex shear modulus is more dominant; a higher score thereby representing that the loss modulus, or imaginary component, is becoming increasingly influential in HC tissue behavior. Accumulating neuroproteomic data demonstrates that hippocampal aging involves common themes of dysregulated metabolism, increased oxidative stress, altered protein processing, and decreased synaptic function (Fan et al. 2017). Taking these findings into consideration, we can speculate that age-related disorganised tissue components may manifest in the MRE signal by being more effective at absorbing strain energy. Alternatively, the mechanism behind alterations in hippocampal ξ have been speculated to potentially relate to neurogenesis (Munder et al. 2017; Schwarb et al. 2017); however, a recent study concluded that neurogenesis does not continue, or is extremely rare, in adult humans (Sorrells et al. 2018). As such, research in animal models of disease will be necessary to allow full interpretation of the neurobiological basis of the MRE signal.

In the future it will be interesting to see if the findings of the present study are replicated and a number of refinements can be introduced. In particular, a limitation of the present study is the inability to rule out any covert neuropathology, even though we made reasonable effort to confirm participants were cognitively healthy. For example, a proportion of older adult participants may have an abnormal amyloid-beta (Aβ) burden that would remain undetected with MRI, even though there is currently no consensus as to whether viscoelastic measures are sensitive to Aβ accumulation (Murphy et al. 2017; Munder et al. 2017). Future studies could also employ a wider range of memory measures and include other imaging biomarkers such as the microstructural measures obtained from diffusion tensor imaging (DTI). DTI can probe the white matter pathways in the hippocampus and has previously revealed a loss of integrity with age (Yassa et al. 2011) and a relationship between hippocampal mean diffusivity (MD) and verbal memory performance (van Norden et al. 2012). The combination of hippocampal MRE measures, MD from DTI, and MR volumetry has recently been shown to improve the diagnostic accuracy of Alzheimer’s disease (AD) (Gerischer et al. 2017). Accordingly, it is conceivable to propose that a combination of imaging modalities that may include MRE could prove useful in the identification of healthy individuals at greatest risk for cognitive decline.

## Conclusions

This is the first report of a significant structure-function relationship between hippocampal viscoelasticity and episodic memory performance in a sample of cognitively intact older adults. Consistent with previous studies of younger adults, greater relative viscous-to-elastic behavior of the hippocampus (i.e. higher damping ratio, ξ) was associated with poorer performance on the verbal paired associates task. In particular, only left hippocampal ξ was associated with recall score, which may be attributed to the verbal nature of the VPA test, thus supporting previous reports of hippocampal functional specialisation. Of note, neither hippocampal volume nor hippocampal stiffness possessed a relationship with task performance. Future research is now recommended to build upon these results in order to establish the causal nature between these variables and whether hippocampal MRE measures can predict future episodic memory decline. Ultimately, understanding how changes in hippocampal microstructure impacts cognition in the context of aging may prove important for identifying intervention targets for combating cognitive aging, and could suggest a possible role for MRE as an imaging biomarker for memory specific disorders such as Alzheimer’s disease.
